# p54nrb/NonO and PSF promote U snRNA nuclear export by accelerating its export complex assembly

**DOI:** 10.1093/nar/gkt1365

**Published:** 2014-01-10

**Authors:** Hiroto Izumi, Asako McCloskey, Kaori Shinmyozu, Mutsuhito Ohno

**Affiliations:** ^1^Institute for Virus Research, Kyoto University, Kyoto 606-8507, Japan and ^2^RIKEN Center for Developmental Biology, Kobe, Hyogo 650-0047, Japan

## Abstract

The assembly of spliceosomal U snRNPs in metazoans requires nuclear export of U snRNA precursors. Four factors, nuclear cap-binding complex (CBC), phosphorylated adaptor for RNA export (PHAX), the export receptor CRM1 and RanGTP, gather at the m^7^G-cap-proximal region and form the U snRNA export complex. Here we show that the multifunctional RNA-binding proteins p54nrb/NonO and PSF are U snRNA export stimulatory factors. These proteins, likely as a heterodimer, accelerate the recruitment of PHAX, and subsequently CRM1 and Ran onto the RNA substrates *in vitro*, which mediates efficient U snRNA export *in vivo*. Our results reveal a new layer of regulation for U snRNA export and, hence, spliceosomal U snRNP biogenesis.

## INTRODUCTION

Major spliceosomal U snRNAs such as U1, U2, U4 and U5 are transcribed in the nucleus by RNA polymerase II (pol II) and acquire an m^7^G-cap structure. In metazoans, U snRNAs are initially exported to the cytoplasm [reviewed in (1)]. U snRNA export requires an m^7^G-cap structure on the RNA (2) and the leucine-rich nuclear export signal (NES) receptor CRM1 (3). The interaction between CRM1 and U snRNA is mediated by two adaptors, the first of which is the nuclear cap-binding complex (CBC), a heterodimeric protein complex consisting of CBP80 and CBP20 (4). CBC binds specifically to the m^7^G-cap structure of nascent RNA Pol II transcripts and promotes U snRNA export as well as pre-mRNA processing and pioneer-round translation [reviewed in (5,6)].

The other adaptor required for U snRNA export is the phosphorylated adaptor for RNA export [PHAX, (7,8)]. PHAX binds to both CBC and the cap-proximal region of U snRNA, resulting in the trimeric ‘pre-complex’. The pre-complex can efficiently interact with CRM1 in a RanGTP-dependent manner, resulting in the ‘U snRNA export complex’. Although the NES of PHAX is essential for the pre-complex to interact with CRM1, this binding is not constitutive, unlike the regular NES-CRM1 interactions (7). Phosphorylation of PHAX by CK2 is essential for this interaction but not for the formation of the pre-complex (7,9). After translocating to the cytoplasm through the nuclear pore complexes, the U snRNA export complex disassembles in a manner that involves both GTP hydrolysis by Ran and PHAX dephosphorylation by PP2A (7,9).

Thus the overall picture of U snRNA export appears to be established. Nevertheless, it is likely that U snRNA export is regulated by various biological signals, e.g. mitogen signals, through regulatory factors that enhance or reduce the efficiency of U snRNA export. Such regulatory factors for U snRNA export are yet to be identified. We previously demonstrated that HeLa cell nuclear extracts (HNEs) exhibited an activity that stimulated PHAX-recruitment to RNA substrates (10). We biochemically characterized this activity in the present study and revealed that RNA-binding proteins p54nrb/NonO and PSF were the responsible factors. p54nrb/NonO and PSF form a heterodimer, which is a multifunctional factor implicated in a wide variety of cellular processes, many of which are related to RNA [reviewed in (11–14)]. A series of *in vitro* as well as *in vivo* experiments with *Xenopus* oocytes and HeLa cells have revealed that these factors, likely as a heterodimer, are U snRNA export stimulatory factors.

## MATERIALS AND METHODS

### Cell culture, antibodies and molecular biology

Human cervical carcinoma HeLa cells were maintained under an atmosphere containing 5% CO_2_ in Dulbecco’s modified Eagle’s medium supplemented with 10% fetal bovine serum, 100 U/ml penicillin and 100 µg/ml streptomycin. Polyclonal anti-PHAX, anti-CBP80 antibodies were as previously described (7). Polyclonal antibody directed against coilin was from Santa Cruz (H-300). Monoclonal anti-hnRNP C, anti-PSF and anti-FLAG tag antibodies were from Sigma (4F4, B29 and M2, respectively). Monoclonal anti-p54nrb and anti-GAPDH antibodies were from BD (3/p54nrb) and HyTest (6C5), respectively. Human p54nrb cDNA was polymerase chain reaction (PCR) amplified from a HeLa cDNA library and cloned into the *Bam*HI and *Xho*I sites of the pGEX-6 p vector. The pET15 plasmid expressing human PSF was a kind gift from Kozo Tomita (AIST). Ftz, U1 and DHFR derivative plasmids were described previously (15). ^32^ P-labeled RNAs were transcribed *in vitro* as described previously (15). RNA was purified from HeLa cells with SepasolTM-RNA I Super (Nacalai Tesque), and then treated with RQI RNase-Free DNase (Promega). Reverse transcriptase-PCR (RT-PCR) was performed with the AccessQuickTM RT-PCR System (Promega). Quantitative RT-PCR (qRT-PCR) was performed with SuperScriptIII Platinum SYBR Green One-Step qRT-PCR (Invitrogen) and a 7500 RealTime PCR System (Applied Biosystems). The nuclear and cytoplasmic RNAs from HeLa cells were prepared as described (16). The U1ΔSm expressing plasmid was constructed as follows.

An *EcoR*V-*Hind*III fragment containing the U1ΔSm sequence was first cloned into the same sites in pUC118 (pUC118-U1ΔSm). The DNA sequence from the human U1 promoter was PCR-amplified from human genome with appropriate primers. The amplified DNA was digested with *EcoRI* and *EcoR*V and cloned into *Eco I*-*EcoR*V site of pUC118-U1ΔSm in the sense orientation (pUC118-hU1promoter-hU1ΔSm). The PCR primers used were as follows (from 5′ to 3′). The GFP-expressing plasmid (pCDNA3-GFP) was previously described (10).

Pre-U1: CAGGGCGAGGCTTATCCATTG, AACTCCAGAAAGTCAGGGGAAAG.

Pre-U4atac: GGCAGTACTGCTAACGCCTG, CTGCTGTTTGAACTGATAAG.

GAPDH: ATGAGAAGTATGACAACAGCCTCAA, AGTCCTTCCACGATACCAAAGTT.

U1 promoter: ACTTACCTGGCAGGGGAGATAC, TCCCCCACTACCAGCTCGAG.

### Recombinant proteins

Recombinant CBC, glutathione S-transferase (GST)-PHAX and hnRNP A1 were prepared as previously described (10). Recombinant CRM1 and Ran and phosphorylated PHAX were prepared as previously described (7). Recombinant human PSF with N-terminal with His-tags was expressed in BL21 (DE3) and purified with Ni-NTA Agarose Beads (Qiagen), according to the manufacturer’s protocol. Recombinant human p54nrb was expressed from pGEX-6 p in BL21 (DE3) and purified with Glutathione Sepharose beads (GE Healthcare). The bound beads were washed three times with Buffer 1 [50 mM Tris–HCl (pH 7.5), 0.5 M NaCl, 1 mM EDTA, 1 mM DTT and 8% glycerol), and were subsequently treated with PreScission Protease (GE Healthcare; 2U/µl in Buffer 1) at 4°C for 4 days. The mixture was spun down and the supernatant was recovered as a purified fraction.

### Purification of the PHAX-recruitment activity

HNE (from 5 × 10^9^ cells; Cil Biotech) was fractionated by ammonium sulfate precipitation, and the pellet from 20 to 35% saturation was collected. The pellet was dissolved in buffer-1 [20 mM Tris–Cl (pH 7.5), 0.1 mM EDTA, 10% glycerol, 6 M urea] and applied to a HiTrap Q HP column (HPLC; GE Healthcare) equilibrated with buffer-1. The bound material was eluted stepwise with 0.2 M, 0.5 M and 1 M NaCl in buffer-1. The fractions containing 6 M urea were slowly dialyzed against buffer-2 [20 mM Tris–Cl (pH 7.5), 0.1 mM EDTA, 0.1 M NaCl, 25% glycerol] at 4°C overnight to refold the protein, which were subsequently dialyzed against buffer-3 (buffer-1 without urea). The active Flow Through fraction was applied to a HiTrap Heparin HP column (HPLC; GE Healthcare) equilibrated with buffer-3. The bound material was eluted with a linear gradient of 0.2–1 M NaCl in buffer-3. The active fractions were applied to a Mono S column (SMART System; GE Healthcare) equilibrated with buffer-3. The bound material was eluted stepwise with 0.1, 0.2, 0.5 and 1 M NaCl in buffer-3. The 0.5 M fraction was applied to a Mono Q column (SMART System, GE Healthcare) equilibrated with buffer-3. The bound material was eluted stepwise with 0.3, 0.5 and 1 M NaCl in buffer-3. The Flow Through fraction was applied to a Mono S column equilibrated with buffer-3. The bound material was eluted with a linear gradient of 0.2–0.5 M NaCl in buffer-3.

### Micro-liquid chromatography tandem mass spectrometry

Micro-liquid chromatography tandem mass spectrometry was performed as described previously (10).

### RNA-protein binding assay

#### GST pull-down assay

GST pull-down assay was performed as previously described (10).

#### Band shift assay

The band shift assay was performed as described (7). In short, ^32^P-labeled m^7^G-capped U1ΔSm was incubated with recombinant proteins in Band Shift Buffer [40 mM Hepes-KOH (pH 7.3), 110 mM KOAc, 6 mM Mg(OAc)2, 250 mM sucrose and 0.8 mg/ml *Escherichia coli* tRNA] for 20 min at 25°C. The samples were then fractionated by native 6% polyacrylamide gel electrophoresis (PAGE) followed by autoradiography.

### Protein–protein binding assay

#### GST pull-down assay

GST pull-down assay was performed as previously described (10).

#### Immunoprecipitation assay

Purified recombinant p54nrb, PSF and CRM1 or Ran proteins (20 μg) were rotated with Protein A Sepharose beads (GE Healthcare), pre-bound with anti-p54nrb, or anti-PSF, or anti-Myc in the presence of 1 mg/ml RNase A in 150 μl of RSB150N buffer [10 mM Tris–Cl (pH 7.5), 150 mM NaCl, 2.5 mM MgCl_2_, 0.1% NP-40]. After the beads were washed three times with RSB150N, the bound material was recovered and analyzed by western blotting.

### FLAG immunoprecipitation assay

Cells were transfected with FLAG-PHAX using Lipofectamine 2000 reagent (Invitrogen). Cells were harvested 24 h after transfection, washed twice with phosphate buffered saline (PBS; 137 mM NaCl, 2.7 mM KCl, 19 mM Na_2_HPO_4_, 2 mM KH_2_PO_4_) and fixed with 1% formaldehyde for 10 min at room temperature. Fixation was quenched in 0.15 M glycine (pH 7.0) for 10 min. After the cells were washed twice with ice-cold PBS, the cell pellets were resuspended in mRIPA buffer [50 mM Tris–Cl (pH7.5), 150 mM NaCl, 1% NP-40, 1 mM EDTA] plus Protease inhibitor Cocktail (Roche). The cell lysates were prepared by sonication with Bioruptor (CosmoBio). The lysates were centrifuged at 14 000 rpm for 10 min at 4°C in a microfuge, and the supernatants were incubated with anti-FLAG M2 affinity gel (SIGMA) for 2 h at 4°C. After the resin was washed three times with mRIPA buffer, the bound material was recovered in sodium dodecyl sulphate (SDS) loading buffer by incubating for 30 min at 70°C, and analyzed by western blotting.

### Microinjection into *Xenopus* oocytes

Preparation of ^32^P-labeled RNAs for microinjection and their microinjection into *Xenopus* oocytes were performed as described previously (7).

### Immunofluorescence cell staining

Cell were grown in eight-well glass chamber slides (Nunc) and fixed with 4% formaldehyde in PBS at room temperature for 20 min. Cells were permeabilized with 0.5% TritonX-100 in PBS for 5 min, washed twice with PBS and blocked with 3% bovine serum albumin in PBS for 30 min. Cells were incubated with primary antibodies at appropriate dilutions at room temperature for 1 h. Cells were washed three times with TBS (40 mM Tris–Cl, pH 7.5, 150 mM NaCl) containing 0.1% Tween-20. Cells were then incubated with Alexa569- or Alexa488-conjugated secondary antibodies. The glass slides were mounted and examined with fluorescence microscopy (Zeiss Axio Observer Z1).

### siRNA-mediated knock down

All siRNAs were obtained from Invitrogen (Stealth siRNAs of the 25-mer duplex). The target sequences (5′–3′) were as follows: GGUGCAUUCCUGAAGUCUCUAAUGU for p54nrb, GGAGGCCCGCCGCCUCCGCCCGCGG for PSF, UUCAUUUCUGGUCUGUUCCCUAGCC for PHAX, GCAUCGAAGUAUUCCGCGUACGAAG for the control siRNA. Transient transfection of siRNAs was carried out using Lipofectamine RNAiMAX reagent (Invitrogen) according to the standard protocol. The U1ΔSm plasmid was transfected using Lipofectamine 2000 reagent (Invitrogen) 48 h after siRNA transfection. Preparation of nuclear extracts from siRNA-transfected HeLa cells was as previously described (17).

## RESULTS AND DISCUSSION

### Characterization of the activity that stimulates PHAX-recruitment to RNA substrates

**Figure 1.**
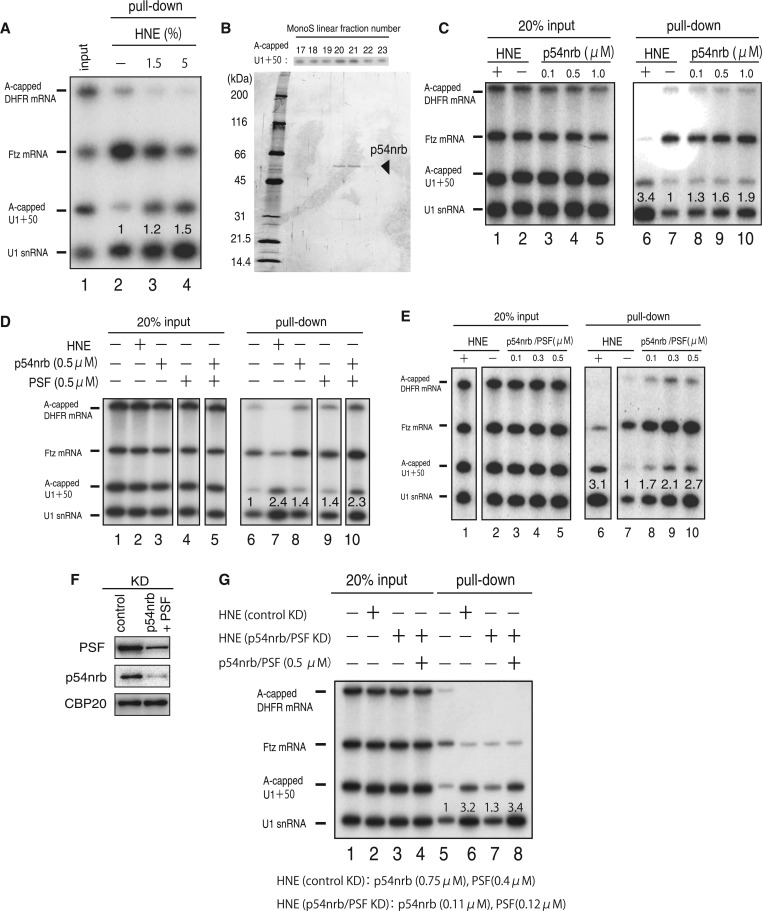
Characterization of the activity that stimulates PHAX-recruitment to RNA substrates. (**A**) A mixture of ^32^P-labeled *in vitro* transcribed RNAs containing A-capped DHFR mRNA, m^7^G-capped Ftz intronless mRNAs (15), A-capped elongated U1ΔSm RNA [U1 + 50 RNAs, (15)] and m^7^G-capped U1ΔSm RNA was mixed with CBC and GST-PHAX or GST (0.2 µM each) and a GST pull-down assay was performed. Co-precipitated RNA was analyzed by 8% denaturating PAGE followed by autoradiography. HNE was added to final concentration of 0–5% in lanes 2–4. Quantification of U1RNA bands in the pull-down panels is shown. (**B**) The final Mono S fractions were analyzed by 4–20% gradient SDS/PAGE and silver staining. An index for the activity is shown on the top. (**C–E**) A GST pull-down assay as in (A) in the absence or presence of recombinant p54nrb, PSF or HNE (15%) as indicated. Quantification of U1RNA bands in the pull-down panels is shown. (**F**) HeLa cells were transfected with siRNAs against p54nrb and PSF and western blotting was performed with antibodies directed against PSF, p54nrb or CBP20 (loading control). (**G**) A GST pull-down assay as in (A) in the absence or presence of HNEs from HeLa cells treated with siRNAs against p54nrb and PSF or with control siRNA, with or without recombinant p54nrb and PSF as indicated. Quantification of U1RNA bands in the pull-down panels is shown. Concentrations of p54nrb and PSF in the corresponding HNEs as measured by western blotting are shown on the bottom.

We previously identified an activity in a HNE that displaces PHAX, the key adaptor protein for U snRNA export, specifically from longer RNAs (10). This led to identification of the hnRNP C tetramer as the factor that removes PHAX from mRNAs, but not from U snRNAs, to maintain the specific composition of each RNA export complex (10). During that identification process, we serendipitously found a separate activity that stimulates PHAX-recruitment to RNA substrates (Figure 1A). *In vitro* transcribed RNAs of various lengths were mixed with recombinant CBC and GST-PHAX fusion protein in the absence or presence of HNE, and a GST pull-down assay was performed to examine the formation of the trimeric ‘pre-complex’ consisting of RNA, CBC and PHAX (Figure 1A). When increasing amounts of HNE were added to the system, PHAX-binding to the longer RNAs was progressively inhibited, while PHAX-binding to the shorter RNAs was progressively stimulated. We previously characterized the former activity (10), and decided to characterize the latter activity in this study because this characterization may lead to identification of regulatory factors for U snRNA export.

This latter activity was purified from HNE through several steps of column chromatography (see ‘Materials and Methods’ section). The final purified fractions contained one major protein band, the appearance of which correlated well with the activity that stimulated PHAX-recruitment (Figure 1B and see Supplementary Figure S1 for the activities monitored during HiTrapQ purification). Using mass spectrometric analysis, we identified this protein as p54nrb/NonO (hereinafter p54nrb), a nuclear RNA-binding protein (11). The recombinant p54nrb protein produced in *E. coli*, however, only weakly stimulated the PHAX-recruitment to the RNA substrates (Figure 1C). In this relation, we noted that a large fraction of activity was lost during the purification step by the HiTrapQ HP column in the presence of 6 M Urea (data not shown). Therefore, we suspected that an important component(s) was lost during the purification process.

Because p54nrb functions, in many cases, as a heterodimer with another RNA-binding protein PSF (11), we considered that PSF may be the missing component. Recombinant PSF alone exhibited a weak activity, as did p54nrb alone (Figure 1D, lanes 8 and 9). However, p54nrb and PSF together exerted much stronger PHAX-recruitment activity (Figure 1D, lane 10, and Figure 1E), which was consistent with the above hypothesis in which p54nrb and PSF function together in this process. Interestingly, the PHAX-recruitment activity was independent of the length of RNA or the presence of the m^7^G-cap structure (Figure 1E). p54nrb and PSF have been implicated in various cellular processes, many of which are related to RNA metabolism (11).

To examine whether p54nrb and PSF constitute the majority of the PHAX recruitment activity in HNE, we tested the activity in the nuclear extract from HeLa cells in which p54nrb and PSF have been knocked down by siRNAs. The typical knock down efficiencies were 85% for p54nrb and 70% for PSF (Figure 1F). The PHAX recruitment activity was greatly reduced in the extract from the knocked down HeLa cells as compared with that from the control cells (Figure 1G, lanes 5–7). Adding back recombinant p54nrb and PSF proteins to the knocked down HNE, to the concentrations comparable with that in the control HNE, fully restored the PHAX recruitment activity (Figure 1G, lane 8). These results strongly suggested that p54nrb and PSF constituted the major PHAX recruitment activity in HNE. The results so far suggested that p54nrb and PSF may be involved in U snRNA export by regulating the recruitment of U snRNA export factors onto U snRNAs, and we decided to test this hypothesis.

### p54nrb and PSF interact with PHAX and stimulate formation of the pre-complex

**Figure 2.**
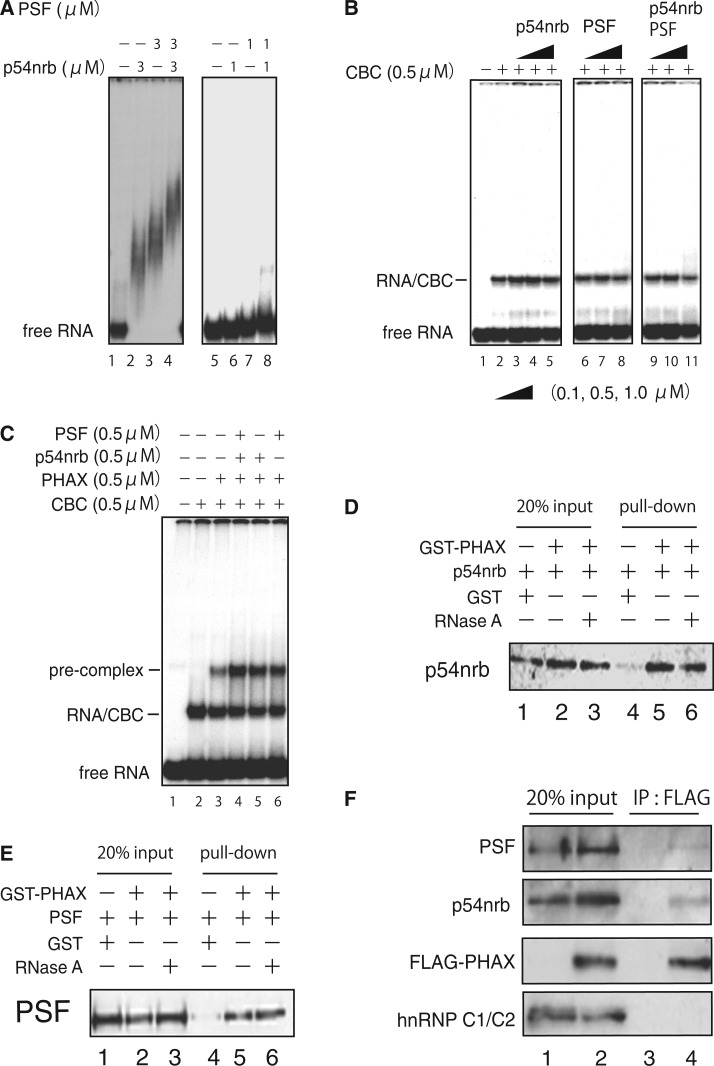
p54nrb and PSF interact with PHAX and stimulate the formation of the pre-complex. (**A–C**) A ^32^P-labeled capped U1ΔSm RNA probe was incubated for 30 min at 25°C in the presence or absence of recombinant CBC, PHAX, p54nrb and PSF as indicated. The samples were fractionated by native 6% PAGE followed by autoradiography. Free RNA probe and major complexes are indicated on the left. (**D**) and (**E**) GST-PHAX or GST (0.2 µM each) was mixed with p54nrb (D) or PSF (E) in the absence or presence of RNase A and a GST-pulldown was performed. The precipitated protein was analyzed by western blotting. (**F**) Flag-tagged PHAX was transiently expressed in HeLa cells. A flag IP was performed and the precipitated protein was analysed by western blotting.

For this purpose, we first used well-established gel-shift analyses to monitor the assembly of U snRNA export-related complexes (7). Both p54nrb and PSF are RNA-binding proteins, and they bound to U1 RNA (Figure 2A). However, their binding affinity to U1 RNA seemed to be low because clear band-shift was observed only at a high protein concentration (Figure 2A). p54nrb and PSF did not stimulate formation of the RNA/CBC complex (Figure 2B), but did stimulate the recruitment of PHAX to the RNA/CBC complex (Figure 2C). Both p54nrb and PSF interacted with PHAX in an RNA-independent manner, as demonstrated by the GST pull-down assay (Figure 2D and E). Moreover, ectopically expressed flag-tagged PHAX could interact with endogenous p54nrb and PSF in HeLa cells, as demonstrated by co-immunoprecipitation experiments (Figure 2F). It is likely that these proteins recruit PHAX onto RNA substrates by simultaneously interacting with PHAX and RNA. However, note that the bands for pre-complex were not clearly super-shifted by the addition of p54nrb and PSF (Figure 2C), indicating that the interactions between these proteins and PHAX, and between these proteins and U1 RNA are unstable.

### p54nrb and PSF interact with CRM1 and Ran, and stimulate the formation of the U snRNA export complex

**Figure 3.**
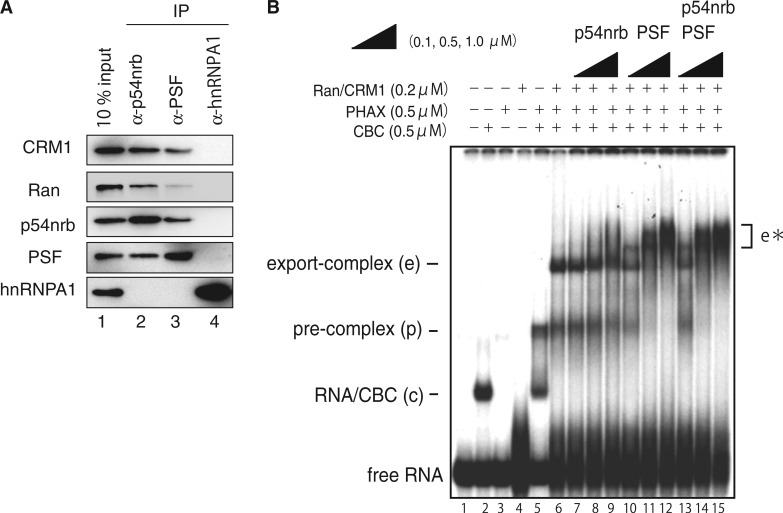
p54nrb and PSF interact with CRM1 and Ran, and stimulate the formation of the U snRNA export complex. (**A**) Recombinant p54nrb, PSF and hnRNP A1 were mixed with recombinant CRM1 or RanGTP. IPs were performed with the indicated antibodies and the precipitated protein was analyzed by western blotting. (**B**) A ^32^P-labeled capped U1ΔSm RNA probe was incubated for 30 min at 25°C in the presence or absence of CBC, phosphorylated PHAX and/or CRM1 plus RanQ69LGTP as indicated. PHAX was phosphorylated by recombinant kinase CK2 (7). The samples were fractionated by native 6% PAGE followed by autoradiography. Free RNA probe and major complexes are indicated on the sides.

Interestingly, p54nrb and PSF could also interact with both CRM1 and Ran (Figure 3A) *in vitro*, whereas control RNA-binding protein, hnRNP A1 could not. P54nrb and PSF could also promote the formation of higher-order complexes *in vitro* (Figure 3B, marked as e*). The smeary e* complexes are likely to be related to U snRNA export because a series of western blotting analyses (Supplementary Figure S2) revealed that they indeed contained known U snRNA export factors such as PHAX, CRM1 and Ran as well as p54nrb and/or PSF. This stimulation of the assembly of export-related complexes (e plus e*) resulted not only from stimulation of the pre-complex assembly. Because the majority of pre-complex bands were super-shifted to the e* complexes in the presence of p54nrb and PSF (Figure 3B), transition from the pre-complex to export-related complexes was indeed stimulated. Unlike pre-complex assembly (Figure 2), the e complex was clearly super-shifted to the e* complexes by the addition of p54nrb and/or PSF. However, the e* complexes were smeary and likely unstable, especially in the case of p54nrb alone (Figure 3B, lanes 7–9). We speculate that p54nrb and PSF may not be stable components of nuclear U snRNA export complexes.

Thus, p54nrb and PSF can interact with PHAX, CRM1 and Ran as well as the RNA substrates; therefore, they are able to load these multiple factors onto the RNA substrates. In fact, p54nrb and PSF were previously shown to interact with a wide variety of other factors including hnRNP M, XRN2 and Topoisomerase I, and appear to work as a general loader (11). Interestingly, this loading activity by itself does not distinguish mRNA from U snRNA. p54nrb and PSF load PHAX onto RNA substrates regardless of their lengths (Figure 1E). However, in the nucleus, this loading activity should be counteracted by the tetramer of hnRNP C, which exclusively binds to and removes PHAX from longer Pol II transcripts, i.e. mRNAs (10). Therefore, as a result, PHAX should remain loaded exclusively on shorter Pol II transcripts, i.e. U snRNAs. Moreover, PHAX is concentrated in the Cajal bodies [CB, (18)], and this localization may increase PHAX's association with U snRNAs that transit CB (19), and decrease PHAX's association with mRNAs that do not transit CB.

### p54nrb and PSF are involved in U snRNA export in living cells

**Figure 4.**
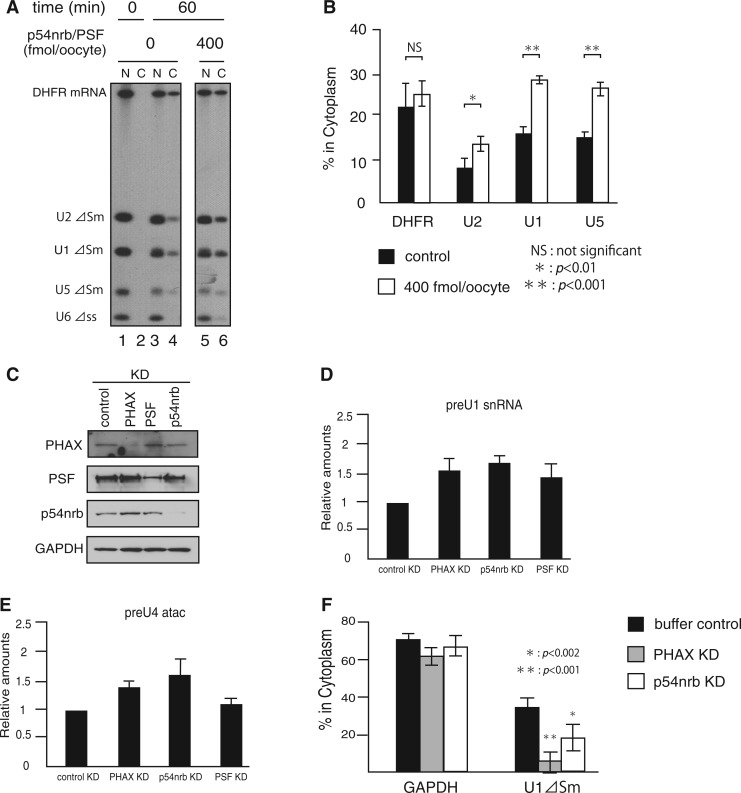
p54nrb and PSF are involved in U snRNA export in living cells. (**A**) A mixture of 32 P-labeled m7G-capped DHFR mRNA, m7G-capped U1ΔSm, m7G-capped U2ΔSm, m7G-capped U5ΔSm and U6Δss RNAs was injected into the nucleus of *Xenopus* oocytes either alone or together with recombinant human p54nrb and PSF (400 fmol/oocyte). RNA was extracted from nuclear (N) and cytoplasmic (C) fractions, immediately (0 min) or 60 min after microinjection and analyzed by 8% denaturating PAGE followed by autoradiography. (**B**) The radioactivity of the bands was quantified from three independent experiments as in (A). The amounts of RNA export (% in cytoplasm) were calculated by taking the U6 RNA leakage into account. The averages and the standard deviations are shown. *P < 0.01; **P < 0.001; NS, not significant. (**C**) HeLa cells were transfected with siRNAs as indicated and western blotting was performed with antibodies directed against PHAX, PSF, p54nrb or GAPDH. (**D**) and (**E**) HeLa cells were transfected with siRNAs as in (C), and qRT-PCR was performed to quantify the endogenous pre-U1 or pre-U4 atac snRNAs. The averages and the standard deviations from three experiments are shown. (**F**) HeLa cells were transfected first with siRNAs as indicated and 48 h later with the plasmid expressing U1ΔSm RNA. After 3 h, RNA was extracted from the nuclear and cytoplasmic fractions of the transfected HeLa cells and examined by qRT-PCR for the nucleo-cytoplasmic distribution of the expressed U1ΔSm RNA or endogenous GAPDH mRNA.

U snRNAs are normally reimported into the nucleus after their initial export (1). The ΔSm mutants of U snRNAs are subjected to the initial export, but not to the subsequent reimport (2). Microinjection of the recombinant human p54nrb and PSF in *Xenopus* oocytes stimulated export of microinjected U snRNA ΔSm mutants, but not of DHFR mRNA (Figure 4A and B), suggesting that these two proteins specifically promoted U snRNA export.

Examining endogenous U snRNA export directly in mammalian cells is not technically possible, mainly because U snRNAs shuttle between the nucleus and cytoplasm and highly abundant mature U snRNAs accumulate in the nucleus. However, the 3′ ends of U snRNA precursors are known to be digested after their initial export to the cytoplasm [reviewed in (1)]. Therefore, the accumulation of longer precursor forms is a good indication of the inhibition of U snRNA export (20). If p54nrb or PSF was knocked down in HeLa cells by siRNAs (Figure 4C), U snRNA precursors significantly accumulated similar to that in PHAX-KD cells, as demonstrated by preU1 and preU4 atac RNAs (Figure 4D and E).

Because the accumulation of pre-snRNAs could result from disruption in other steps of snRNP biogenesis in addition to U snRNA export, we also tested the efficiency of U snRNA export more directly in HeLa cells (Figure 4F). A plasmid expressing U1ΔSm RNA was transfected to HeLa cells, and the efficiency of U snRNA export was assessed by calculating the cytoplasmic to nuclear ratio (C/N) of U1ΔSm RNA after cells were fractionated into the nuclear and cytoplasmic fractions. U snRNA export was significantly reduced in p54nrb-KD cells, similar to PHAX-KD cells. We could not examine PSF-KD cells because of their extensive death. Taken together, these results indicated that p54nrb and PSF promoted U snRNA export in living cells.

### p54nrb and PSF do not accompany UsnRNAs to the cytoplasm

Whether p54nrb and PSF accompany the transported RNA into the cytoplasm has yet to be established. However, they may not do so because these proteins do not seem to be stable components of nuclear U snRNA export complexes. We next examined this issue.

**Figure 5.**
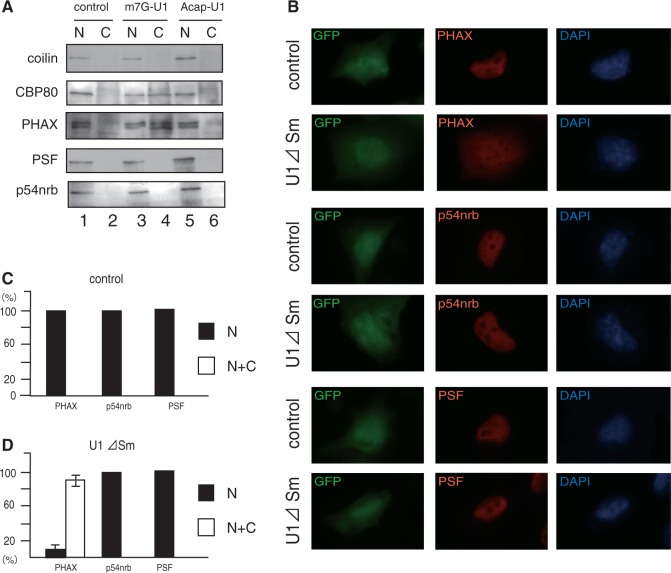
p54nrb and PSF do not accompany U snRNAs to the cytoplasm. (**A**) Three hundred fmol/oocyte of either m^7^G-capped or A-capped U1ΔSm RNA was injected into the nucleus of *Xenopus* oocytes. Twenty hours after injection, the injected oocytes were fractionated and the protein was analyzed by western blotting using the antibodies indicated. Uninjected oocytes were used as a control. (**B**) HeLa cells were transfected with either the vector (control) or the plasmid expressing U1ΔSm RNA (U1ΔSm) together with a GFP-expressing plasmid (pCDNA3-GFP) to mark transfected cells, and after 24 h, endogenous p54nrb, PSF and PHAX were localized by immunofluorescence staining in GFP-positive cells. DAPI was used to visualize DNA. (**C**) and (**D**) Quantitative analyses of the localization of PHAX, p54nrb and PSF with U1ΔSm RNA overexpression in the similar experiments as in (B). The percentage of cells with immunofluorescence signals exclusively in the nucleus (N) or in both the nucleus and cytoplasm (N + C) were calculated. One hundred GFP-positive cells were examined.

The steady state localization of CBC and PHAX in *Xenopus* oocytes is mainly nuclear [Figure 5A, lanes 1, 2, also see (7)]. However, significant fractions of CBC and PHAX were previously shown to move to the oocyte cytoplasm when a saturating amount (21) of m^7^G-capped U1ΔSm RNA was injected into the nucleus [Figure 5A, lanes 3 and 4, also see (7)]. This finding shows that CBC and PHAX move to the cytoplasm together with U snRNA and subsequently recycle back to the nucleus alone (7). We analyzed the behaviors of microinjected human p54nrb and PSF in the same experimental system (Figure 5A, lower two panels). Neither protein moved to the cytoplasm under the conditions that induced efficient export of CBP80 and PHAX although these proteins stimulated U snRNA export (Figure 4A and B).

To confirm these results in the mammalian cell system, U1ΔSm RNA was overexpressed in HeLa cells by transfection of an expression plasmid, and the endogenous PHAX protein was localized by immunofluorescence cell staining. Under control situations, PHAX was mainly nuclear as expected [Figure 5B, control panels and Figure 5C, (8)]. However, when U1ΔSm RNA was overexpressed, a fraction of endogenous PHAX moved to the cytoplasm (Figure 5B, U1ΔSm panels, and Figure 5D). p54nrb and PSF showed nuclear staining with some dot structures corresponding to para-speckles (12–14) under the control situations (Figure 5B, control panels, and Figure 5C) and these staining patterns persisted even when U1ΔSm was overexpressed (Figure 5B, U1ΔSm panels, and Figure 5D), unlike the case of PHAX. These results suggested that p54nrb and PSF are likely to fall off the U snRNA export complex before or during translocation through the nuclear pore complexes.

In this study, we have described the identification of p54nrb and PSF as U snRNA export stimulatory factors. The way they work is by stimulating the loading of U snRNA export factors onto the RNA substrates. p54nrb and PSF can interact with PHAX, CRM1 and Ran as well as the RNA substrates; therefore, they are able to load these multiple factors onto the RNA substrates. After loading the export factors, p54nrb and PSF are likely to fall off the U snRNA export complex, and therefore they are able to participate in the next round of loading. Thus p54nrb and PSF chaperone the U snRNA export factors onto the RNA. We speculate that p54nrb and PSF could be targets for biological regulations of spliceosomal U snRNP biogenesis.

## SUPPLEMENTARY DATA

Supplementary Data are available at NAR Online.

## FUNDING

Grant-in-Aid for Scientific Research on Innovative Areas ‘Diversity and asymmetry achieved by RNA RNA program’ [No. 20112007 to M.O.] from the Ministry of Education, Culture, Sports, Science and Technology (MEXT) of Japan. Funding for open access charge: Grant-in-Aid for Scientific Research on Innovative Areas ‘Diversity and asymmetry achieved by RNA RNA program’ [No. 20112007].

*Conflict of interest statement*. None declared.

## Supplementary Material

Supplementary Data
