# Supplementary material for: p54nrb/NonO and PSF promote U snRNA nuclear export by accelerating its export complex assembly
Source: Nucleic Acids Res. 2014 Jan 10;42(6):3998–4007. doi: 10.1093/nar/gkt1365 (PMC3973303; doi:10.1093/nar/gkt1365)
Supplement: Supplementary Data [file supp_42_6_3998__index.html]

p54nrb/NonO and PSF promote U snRNA nuclear export by accelerating its export complex assembly — p54nrb/NonO and PSF promote U snRNA nuclear export by accelerating its export complex assembly — Supplementary Data 

# p54nrb/NonO and PSF promote U snRNA nuclear export by accelerating its export complex assembly

## Supplementary Data

files

**Files in this Data Supplement:**

- Supplementary Data - pdf file
