# Supplementary material for: p54nrb/NonO and PSF promote U snRNA nuclear export by accelerating its export complex assembly
Source: Nucleic Acids Res. 2014 Jan 10;42(6):3998–4007. doi: 10.1093/nar/gkt1365 (PMC3973303; doi:10.1093/nar/gkt1365)
Supplement: Supplementary Data [file supp_gkt1365_nar-02823-a-2013-File007.pdf]

Figure S1

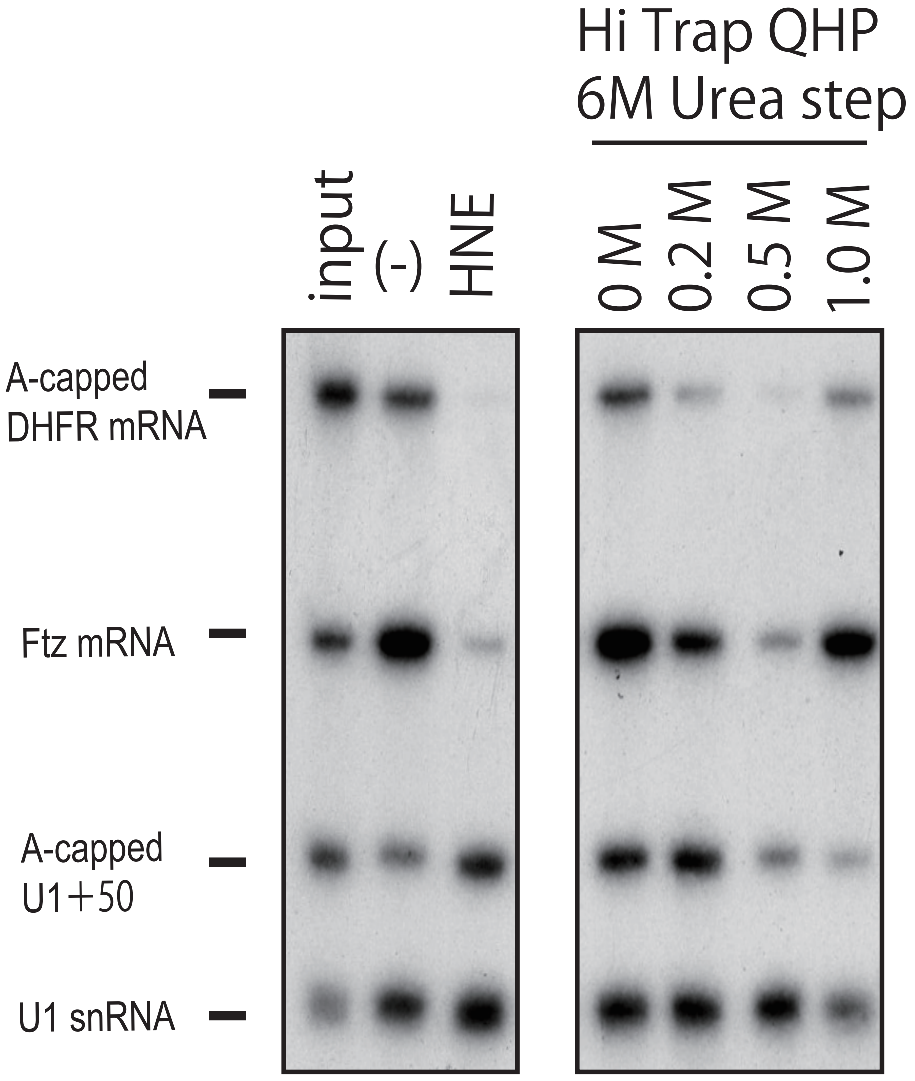

Fig.S1 Purification of the activity that stimulates PHAX-recruitment to RNA.
A GST-PHAX pull-down assay similar to that in Fig.1A was performed with the fractions from HiTrapQ HP column chromatography in the presence of 6M Urea.

Figure S2

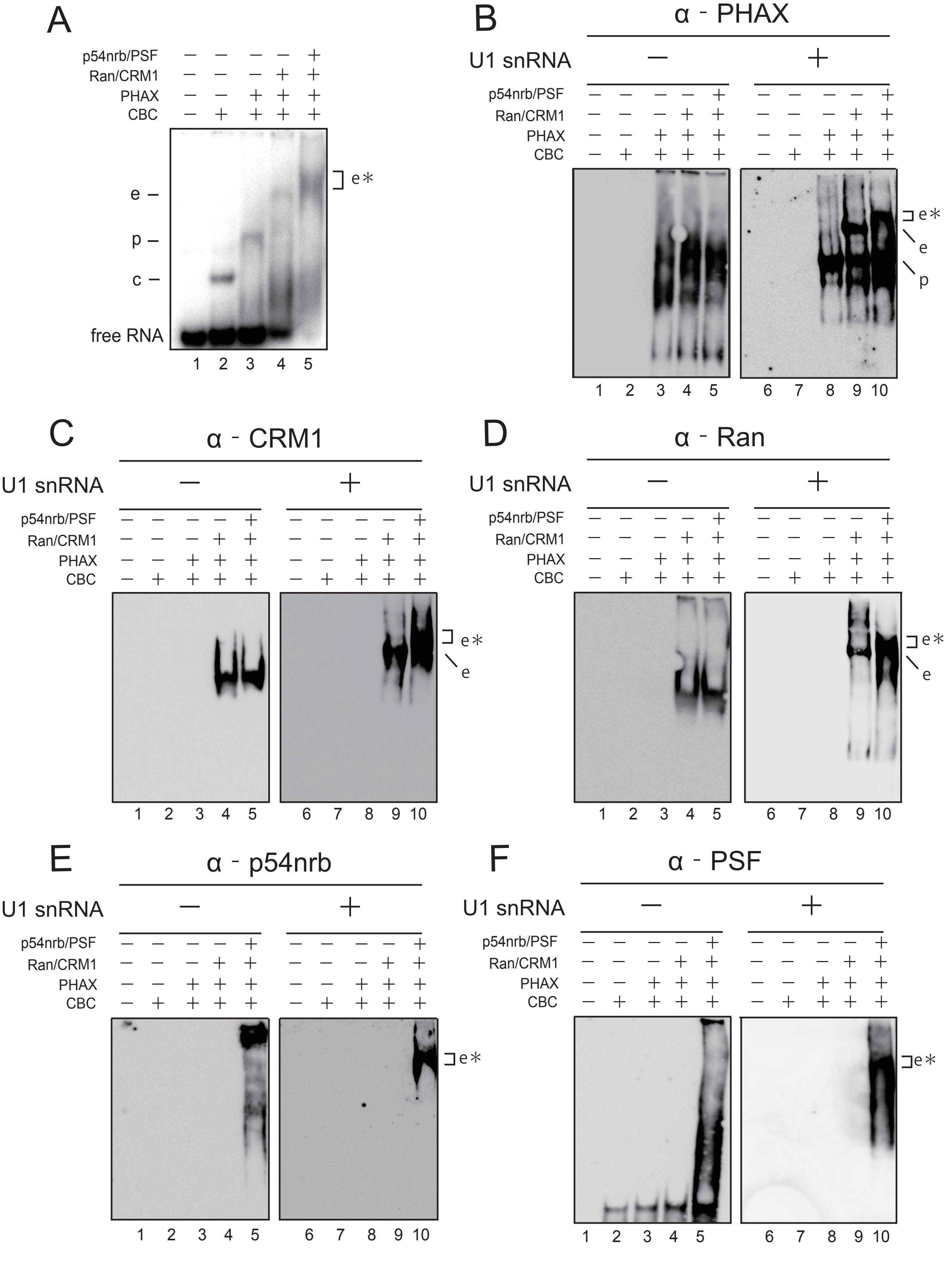

Fig.S2 (A) A similar band shift analysis as in Fig.3B except that cold capped U1ΔSm RNA (1μg/lane) was also added. (B)-(F) The gel from the same experiment as in (A) was blotted onto a nitrocellulose membrane and western blotting was performed with the indicated antibodies.
